# Wnt signaling receptor LRP6 interacts with amyloid precursor protein and regulates its trafficking and processing

**DOI:** 10.1186/1750-1326-7-S1-O5

**Published:** 2012-02-07

**Authors:** Chia-Chen Liu, Hyang-Sook Hoe, Guojun Bu

**Affiliations:** 1 Department of Neuroscience, Mayo Clinic Jacksonville, Florida, USA; 2 Department of Neuroscience, Georgetown University, Washington, D.C., USA

## 

The processing of amyloid precursor protein (APP) to Aβ is an important event in the pathogenesis of Alzheimer’s disease (AD). Previous studies suggest that dysregulation of Wnt signaling is involved in the etiology of AD. Genetic variation in the Wnt receptor, low-density lipoprotein receptor-related 6 (LRP6), which is associated with reduced Wnt signaling has been reported as a risk factor for AD. In the present study, we found that LRP6 expression is down-regulated in AD brains. LRP6 and APP colocalized in neurons and interact with one another through their extracellular domains. LRP6 overexpression increases cell surface levels of APP and decreases Aβ production, while RNA interference of LRP6 expression increases APP endocytosis and Aβ levels. These results demonstrate that LRP6 plays an important role in the regulation of APP trafficking and processing to Aβ. Our findings suggest that rescuing LRP6-mediated Wnt signaling might be an attractive therapeutic strategy for AD.

